# Redemption from plight: a qualitative study on reasons behind treatment decisions among Iranian male opioid users

**DOI:** 10.1186/s13011-020-00299-x

**Published:** 2020-08-08

**Authors:** Mostafa Amini-Rarani, Esmaeil Khedmati Morasae, Tahereh Pashaei, Maryam Moeeni

**Affiliations:** 1 Health Management and Economics Research Center, Isfahan University of Medical Sciences, Isfahan, Iran; 2 Centre for Circular Economy, Business School, University of Exeter, Exeter, UK; 3 Environmental Health Research Center, Research Institute for Health Development, Kurdistan University of Medical Sciences, Sanandaj, Iran; 4 Social Determinants of Health Research Center, Isfahan University of Medical Sciences, Isfahan, Iran, Hezar-Jerib Ave, Isfahan, 81746 73461 Iran

**Keywords:** Opioid-related disorders, Patient preference, Motivation, Thematic analysis, Iran

## Abstract

**Background:**

Opioid use remains a significant cause of harm to individual health. Perceived motives are of the main factors that help lead a patient into seeking treatment voluntarily to obviate that harm. The current study expands on the literature by exploring when and how male users of opioids become motivated to voluntarily seek treatment services.

**Methods:**

In a qualitative study in Isfahan city from January 2018 to March 2019, 55 male participants who had already started a variety of treatment services to withdraw their dependence on opioids were recruited. Selection of participants was based on a maximum variation purposive sampling strategy. Each participant took part in a unstructured interview to identify his motives for seeking opioid use treatment. Interviews were undertaken in eight different treatment centers. An inductive thematic analysis method was used to analyze the interviews.

**Results:**

The findings highlight that Iranian male opioid users have different motivations to seek treatment. To be precise, the findings illuminate three global themes and six themes as treatment-seeking motives among the participants including; motives related to family (reason *for* family and reason *of* family), quality of life (adverse effects on personal lifestyle and health) and economic motives (financial failure and job failure).

**Conclusions:**

The findings can improve our understanding of the motives for seeking treatment from the perspective of opioid patients who entered themselves into treatment. Particularly, these findings could help policymakers and treatment providers to better understand opioid-use patient’s perceived concerns and fears as motives for treatment-seeking.

## Background

The International Classification of Disease (ICD-10) has classified opioid use disorder (OUD) as a chronic and relapsing disorder [1]. The World Drug Report estimated 53 million opioid users worldwide in 2017, which is up 56% from previous estimates [2]. The opioid use crisis remains a significant cause of individual and public health harms, affecting over 130 million people worldwide [3, 4]. As such, it has been the main contributor to morbidity and premature mortality resulting from an overdose, suicide, trauma, and blood-borne infections such as HPV and hepatitis as well as a major risk factor of HIV epidemics worldwide [5–8]. In total, opioids were responsible for two-thirds of 585,000 death numbers due to drug use throughout the world in 2011 [9]. They also accounted for 9.2 million worldwide Disabilty Adjusted Life Years (DALYs) that were attributed to substance use disorders in 2010 [10]. Aside from physical harm, previous studies also have shown that opioid users were at higher risk of panic, social phobia, agoraphobia, and low self-reported mental health as well as anxiety and mood disorders [11–13]. Also, OUDs incur significant societal costs including healthcare burden, reduced productivity, drug enforcement cost, and crime [9, 14, 15]. Appropriate treatment interventions for patients with substance use disorders have shown promise in the improvement of physical and mental health, rehabilitation of social communications, and reduction in new substance users [16–18]. Opioid patients who join the treatment programs earlier usually show better outcomes [19]. There are different mechanisms that bring patients with substance use disorders into treatment services. Perceived motives are of prominent mechanisms that can help lead patients to seek treatment voluntarily [16, 20, 21].

Iran has one of the highest rates of opioid use globally. Moreover, OUD is the most prevalent type of drug use disorder in Iran [22], estimated to be 1.8% among people aged 15–64 in 2011 [9]. Opium has been the most popular opioid-type drug used traditionally in the country since long ago [5, 23], but other types of opioids have also entered Iran’s illegal drug market in recent decades [22, 24]. In a study among drug users in Tehran, it was shown that opioids and stimulants were the predominantly used drugs [4]. More importantly, opioid use is more prevalent among men in Iran [22, 25, 26] Although substance use has increased significantly among women in recent years, there is evidence that men still show a much higher rate (8 to 10 times higher than the rate among women) [25, 27].

A growing body of literature emphasizes the motives of treatment-seeking are of high importance in the treatment process [26, 28–31]. This matter has led to discussions about motive-based interventions [21, 30, 32]. Clinicians, on the other side, view motives as a critical factor to improve treatment outcomes, mainly in terms of retention and relapse [30, 33, 34].

There have been several studies so far to reveal the reasons behind the engagement of OUDs patients in treatment programs. To the best of our knowledge, while some of these studies focused on features of treatment programs as motives for treatment commencement [24, 29, 35–39], few investigated the OUDs associated problems as motives for treatment-seeking [5, 17, 40]. The present study is among the few studies to explore reasons different from treatment programs’ features as motives for treatment-seeking among OUD patients in Iran. This qualitative study, therefore, contributes to the literature by exploring when and how male users of illicit opioids decide to voluntarily seek treatment. The importance of such qualitative studies for a deeper understanding of treatment-seeking among substance users is noted in some studies [41, 42].

## Methods

### Study design

We used an exploratory qualitative study [43] in which 55 male participants who had already begun a procedure to withdraw their dependence on opioids were recruited. Overall, our study activities were primarily qualitative and focused on deepening our understanding of treatment-seeking among OUD patients in Iran.

### Study setting

Isfahan city, the capital of Isfahan province, is located in the center of Iran. Isfahan is home to around 1.6 million people and is the third-largest city in the country. According to the best of our knowledge, there was a public residential treatment center, a private inpatient center, and two public methadone maintenance treatment (MMT) centers in the city at the time of the study. In our sampling, we included all (i.e. four centers) of the treatment centers mentioned above. Moreover, a couple of private residential treatment centers (called camp), private MMT centers, and several drop-in centers (DIC) were available in this city as well. As a result, by adopting a convenience sampling method, we selected two private MMT centers, a private residential treatment center, and a DIC as our four other sampling sites.

### Population sample

A total of 55 OUD patients were interviewed. The selection of OUD patients was purposive. Adopting a maximum variation purposive sampling strategy, we aimed to find the relevant interviewees from a sampling frame of all the OUD patients who visited the selected treatment centers. The maximum variation approach was adopted to select a sample of respondents with a diverse range in terms of age, education, and lifetime OUD status. This approach could also lead to more robust insights in terms of motives for treatment-seeking from the OUD patients’ perspectives. The inclusion criteria were as follows: 1) being an OUD patient for at least 1 year prior to the interview; 2) being over 18 years old; 3) entering the current treatment program voluntarily; and 4) being mentally and physically able to participate in the face to face interviews. In each selected center, an informed staff helped us to find a list of patients who met the inclusion criteria. Then, a maximum variation sampling method was used to choose the final sample from the eligible patients. The number of people interviewed at the 8 selected centers was as follows: 9 patients in the public residential treatment center, 8 in the private residential treatment center, 4 in the private inpatient center, 11 in the two public MMT centers, 19 in the two private MMT centers, and 4 in the DIC.

### Data collection

Data were gathered through unstructured interviews (*n* = 55). Interviews were conducted in a private room inside the treatment centers, recommended by the centers’ executives. Participation in the interviews was voluntary. To build a rapport with patients, interviewers started the interview sessions with an introduction to the purpose and format of the interview. The participants were also informed and assured that their privacy and confidentiality will be preserved in all stages of the research. Upon acceptance to participate in the interview, each participant was requested to provide verbal informed consent. To maximize the participants’ anonymity, the interviewers avoided asking patients’ family names. Our study was also approved by the ethical committee at Isfahan University of Medical Sciences.

Each participant took part in an unstructured interview. At first, the participants were asked a general question: “why did you decide to treat your disease? Follow-up questions were then used to extract detailed answers to the general question. The interviewer developed, adapted and produced follow-up questions reflecting the central purpose of the study.

Two members of the research team (MM and MAR) conducted face-to-face interviews in a comfortable and quiet room in the treatment center. The principal interviewer (MM) observed all the interviews conducted by the other interviewer (MAR). Both interviewers have enough knowledge and experience in terms of qualitative studies and interviewing, which would help maintain consistency across all interviews. However, to ensure consistency between the interviewers, interviews were conducted based on a topic guide developed according to the study objective and informed by a literature review. Five pilot interviews were also conducted to practice the interviewing. Besides, both interviewers participated in the data analysis, providing them an opportunity to become more consistent over the data collection process.

The interviews were carried out in local language (Persian), audio-taped and continued until the point of saturation. Data saturation was achieved after 55 interviews. The fieldwork and data analysis took place simultaneously from January 2018 to March 2019. Interviews generally lasted up to 40 min.

### Data analysis

An inductive thematic analysis approach was used to identify, analyze and report patterns (themes) within the qualitative data [44]. All the audio-recorded interviews were transcribed verbatim and entered into the MAXQDA Plus (version 12) for analysis. The use of a computer-aided qualitative data analysis tool such as MAXQDA in qualitative research means that the data analysis is carried out in a continuous and consistent manner [45]. The thematic analysis was conducted in accordance with recommendations suggested by Braun and Clarke [44] to develop a set of initial codes, sub-themes, themes and, ultimately, global themes. Firstly, one author (MAR) immersed himself into the data by listening to recorded interviews and reading through the interview transcripts in order to get familiar with the data. The interview transcripts were coded after this immersion into data. The initial codes were defined inductively based on the semantic characteristics of the data. The initial codes were later verified and reviewed by second researcher (MM) who tried to discover themes through combining several initial codes. At the end of this stage, a set of themes were created and named. In the next step, all the global themes, themes and sub-themes were re-visited and refined in a bilateral meeting in which inconsistencies were discussed and resolved. Any disagreement at this stage was then discussed and resolved through consultations with two other authors (EKM and TP). Eventually, the final global themes, themes and sub-themes resulting from this refining process were agreed upon and a scholarly report of the analysis was produced.

A qualified bilingual (Persian-English) co-author (EKM) specialized in qualitative research has made a significant contribution to the translation process to address translation problems, mainly translation of quotations and their consequent loss of meaning. We also sent the manuscript to a native English editor to ensure that the standards of the academic language would be guaranteed in the translation process.

### Quality assurance

To secure the quality of the study, four following trustworthiness criteria were observed [46]. 1) Credibility was addressed by prolonged engagement (i.e, 15 months) and triangulation of all researchers in the study. Multiple perspectives of the researchers led to a satisfactory level of depth and breadth to the subject of the study. For instance, any conflicting issues in coding and the thematic process were deeply discussed and resolved.

2) Transferability was enhanced by selecting OUDs through a purposeful sampling method, providing a thick description of the findings. Moreover, data collection and analysis were also conducted simultaneously. 3) Dependability of the research was achieved through an audit trail in which the peers accompanying two external auditors engaged in complementary comments on the coding process, analysis of the interview transcripts, and cross-checking of collected data. 4) Confirmability was established by not involving the personal values and theoretical orientations of the research team in the research process.

## Results

Descriptively speaking, participants were on average more than 37 years old and had been an opioid user for more than 12 years. Most participants were married and graduated from high school. The participants would use at least one type of opioid including opium, heroin, Asian crack (a heroin based substance), opium juice, methadone, and tramadol. As Table 1 shows, 23 participants (next to 42%) were multi-users who used at least two types of opioid-based drugs together; among them, about 35% used opium and heroin.

**Table 1:** Demographic and therapeutic features of participants in the study

Features	Mean (SD)/Frequency (%)
Age	37.60 (10.06)
OUD duration (year)	12.43 (7.19)
Education level	
Primary school	10 (18.18%)
Less than a high school diploma	16 (29.10%)
High school graduate	20 (36.36%)
University	9 (16.36%)
Marital status	
Married	30 (54.55%)
Single	23 (41.82%)
Divorced	2 (3.63%)
Being a father	21 (38.18%)
Multi-user	
Opium & heroin	8 (34.78%)
Opium & opium juice	4 (17.39%)
Opium & opium juice & heroin	2 (8.70%)
Heroin & Asian crack	2 (8.70%)
Others	7 (30.43%)

According to the analysis of the transcripts, there were three global themes as the motives behind the treatment-seeking for opioid use: motives related to family, motives related to the quality of life, and economic motives. Each of these motives had its themes as follows. The themes under the familial motives were “reason for family” and “reason of family.” The themes under motives related to the quality of life were “adverse effects on personal lifestyle” and “adverse effects on personal health.” The themes under economic motives were “financial failure” and “job failure.” These themes also had their sub-themes, which are shown in Table 2. Each of the themes will be discussed in more detail in the following paragraphs.

**Table 2:** Elicited global themes, themes, and sub themes

Global theme	Theme	Sub-theme
Familial motives	Reason for family	Family annoyance
		Family disgrace
		A negative role model for children
	Reason of family	Upsetting family relationships
		Family encouragement
		Family building
Life quality motives	Adverse effects on personal lifestyle	Lifestyle changes
		Dishonor
		Waste of life
		Homelessness
		Symbolic addict
		Crime avoidance
	Adverse effects on personal health	Physical and mental disorders
		Impure drug
		Lack of euphoria
		Injection problem
Economic motives	Financial failure	Waste of wealth
		waste of money
	Job failure	reputation loss
		job loss

### Familial motives

The emergence of family-related themes from the transcripts indicates of the unique place of family concept in issues related to OUD, treatment decision in this case, in the Iranian culture. Two main themes that emerged from the interview transcripts pointed to the roles that negative familial impacts of OUD (reasons for family) and positive effects of family members and issues (reason of family) played in encouraging participants to take part in treatment programs.

Reason *for* family, which consists of three sub-themes of “family annoyance,” “family disgrace,” and “a negative role model for children,” refers to attempts in which the person entered the treatment as they observed how their families were negatively affected by their dependence to opioids and aimed to prevent them from annoying and disgracing the families more. For instance, in referring to this reason, one participant said:

*“The main reason for me was that I had become the disgrace of my family … Keeping my family’s face and grace was hugely important for me since my family is honorable.”*(Participant 8).

This quote was chosen to show the importance of family grace for participants. According to some participants, drug use was seen as a disgrace to their family and the feeling that they were the source of such a disgrace motivated them to enter treatment programs.

Another participant stated the following:

*“My second reason is because of my daughter. She is 14 years old and will get married after some years in the future. Won’t she? I don’t want her to be rebuked then because of my addiction.”* (Participant 6).

A sense of disgrace and annoyance to the family can be understood from this quote as well. Some participants believed that almost all of their family issues were negatively affected by their drug use disorder, in this case, the chance of getting married in the future. Therefore, to prevent from having negative impacts on family members’ lives and future, they sought treatment.

Reasons *of* family, which include “upsetting family relationships,” “family encouragement,” and “building and having a family” sub-themes, points to the role that family members, risk of upsetting familial relationships and thinking of marriage had in encouraging the participants to take part in treatment procedures. For example, in referring to such a role for family, one participant said:

*“We were in the house one day and my wife suddenly told me: Ali, you have been up in the space for a long while, come down to earth for another while please, and see what it is like to withdraw opium? See which one is better, being free from opium or this addiction? Let’s withdraw for a while and if it is a bad experience, then you can get back to addiction and I will have no problem with it then … but thank God … it was a very good experience, my wife helped me and encouraged me to withdraw.”* (Participant 46).

This quote was selected to show how family members’ encouragement was important in motivating some participants to seek treatment. Family members pushed, encouraged, and stayed along with some participants throughout the treatment decision and process. In this example, for instance, the partner acted as the source of treatment motivation for the participant.

Another participant said:

*“It is for my family that I want to withdraw. I was losing them. They told if you continue using drugs, they would leave me, would file a divorce application …*” (Participant 27).

This quote shows the way drug use disorder did put the integrity of some participants’ families at risk and why the fear of losing integrity encouraged some to seek treatment. This quote is in contrast to the previous one where the family stayed for the participants and helped them to go through the treatment process, while in this case the family threatened to break-up and this acted as the motivation.

Another participant emphasized the importance of having a family:

“*… because I like it as well, you know, I should get married, I want to get married, I want to start a family. This is my natural right to build a family of my own.”* (Participant 28).

According to this quote, the tendency to build a family was a strong motive for some participants to enter the treatment programs. The quote also indicates that two issues were important for participants: the marriage itself that is seen as a sacred practice and commitment in the Iranian culture; and the fact that drug use cannot act as a deterrence against personal aspirations and treatment can be a way to fulfill those aspirations.

### Life quality motives

Themes related to the negative personal impacts of OUD were the most common reasons for treatment-seeking among the majority of participants. Adverse effects on personal lifestyle, which is made up “lifestyle changes,” “dishonor,” “waste of life,” “homelessness,” “symbolic addict,” and “crime avoidance” sub-themes, refers to effects that OUD had on participants’ physical appearance, social face (dishonor), behavior, purposefulness, way of living (homelessness), earning (crime-oriented), and enjoyment that were all opiate-centered. These effects acted as motives for seeking treatment. To provide a better description of this sub-theme, three quotes from the participants were as follows:

*“Another thing is that when I was addicted to opium, my wife kept telling me that it stands out a mile that you are a junkie, so try not to go on streets, try to reduce your social appearance, … and these made me think of treatment.”* (Participant 51).

This quote points to the fact that as OUD and its unsightly effects were seen as a taint and dishonor in the social circle around participants, some of them were urged to seek treatment to gain their social face back.

*“I see my personality as a person who is not able to commit robbery, but sometimes you have no choice … the hangover was pushing me towards that way … I was little by little getting to that point to commit robbery … my main reason was this warning alarm, how close I was to that point … the moment I saw this alarm is ringing and I am going to be like this junkie person, that junkie, and I cannot be like them, I decided to withdraw.”* (Participant 29).

Getting closer to a criminal-like lifestyle where participants had to commit robbery to afford their opium costs, as the above quote illustrates, was a personal motive for some participants to seek treatment. In fact, a self-image that some participants had about themselves, even when they were dealing with the problem of drug use, was in accord with criminal avoidance and any danger to that self-image was a motive for them to act and resolve the main problem, i.e. OUD.

*“The time that I spent on addiction, if I had spent on something else, I would have obtained a Ph.D. in it.”* (Participant 50).

This quote was chosen to illustrate the way some participants were thinking of the huge toll of OUD on their life. For instance, the concern that OUD swallowed a huge chunk of their lifetime and it was not worth it acted as a motive for some people to seek treatment.

In addition to above-mentioned personal impacts of OUD as motives for seeking treatment, adverse effects on the personal health of the OUD, which includes the sub-themes of “physical and mental disorders”, “impure drug” “lack of euphoria” and “injection problems” were also raised as personal motives to withdraw opiates. These themes cover participants’ concerns about the effects of OUDs on their physical and mental well-being. The following quotes are two examples of such concerns:

“*… because I did not want to die from addiction. I have seen quite many of my doper comrades who died of this. For example, one of my neighbors died before his twentieth, he died from syncope, just when he was around 16 to 18 years old … I saw these and got to this conclusion that, hey dude, death is the final stop of this route.”* (Participant 35).

*“I had become depressed, dissociable, and …*” (Participant 37).

These two quotes were chosen to illustrate the repercussions of OUD health effects on participants’ motivation to seek treatment programs. As one of the quotes shows, observing other people’s ill health and death due to OUD, and fear of the same destiny, was a big mental blow to some participants to start treatment. For some other participants, their health problems induced by OUD (e.g. depression and injection-related damages) provided the power to leave behind the plight of OUD.

### Economic motives

Alongside familial and life quality motives, economic motives were the next global theme of motivation for treatment-seeking. This global theme included two themes of “financial failure” and “job failure” that will be fully addressed in the next paragraphs. Financial failure, which includes “waste of wealth” and “waste of money” as its sub-themes, denotes how OUD destroyed the financial goals of participants and was identified as an important concern that could persuade some patients to go for treatment. The following quotes are examples of such concerns:

*“Well, I had experienced the sobriety … I tasted and experienced it for nine years; oh my God, it was good, my life was back on track and plan and I had become rich, I built a house, bought a car, I achieved all of these.”* (Participant 2).

*“If I have at least saved the money that I gave away (for drugs), couldn’t it be a huge fortune?”* (Participant 50).

*“My main reason! You know, for 20 years I have been working and making money and giving it away to drug dealers!”* (Participant 39).

All these three quotes illustrate how the plight of OUD jeopardized and destroyed the financial status of the participants and the way the realization of this issue pushed some participants to stop the OUD and, as a result, lose more money. Interestingly, some participants, e.g. the person in the first quote, experienced the sobriety for a while and saw how their financial status improved dramatically and this kept him on track of treatment-seeking.

Job failure, which has “reputation loss” and “job loss” as its sub-themes, refers to the negative effects of OUD on participants’ reputation and occupational opportunities. These negative occupational experiences were the main motives for some participants who sought treatment services. The following quotes illustrate some of such occupational experiences.

“*I am a driver; passengers’ lives are in my hands, I was afraid that addiction will finally cost me dear. We have to renew our documents annually and I was afraid that they will stop qualifying me then.”* (Participant 46).

This quote is quite interesting as it shows how job-related issues were important for some participants in terms of treatment decisions. For this person, for instance, the necessity of driving documents renewal and the fact that he was responsible for the safety of his passengers encouraged him to seek treatment. A sense of job insecurity can be captured from this quote as well, an issue that was also a concern for some other participants.

*“Another reason was that I was a shopkeeper and I lost my shop because of this (OUD). Costs aside, I lost my reputation as a shopkeeper.”* (Participant 48).

This quote is quite interesting because it shows that why not only loss of a job, but losing the job-related reputation made some participants re-think their OUD status and aim for treatment seeking. This quote was chosen to show how dynamics of job and reputation emerged in the manuscripts as determinants of the treatment decision.

## Discussion

This qualitative study explored how perceived concerns and fears related to drug use disorder acted as motives for treatment-seeking among opioid-use male patients voluntarily attending a variety of treatment centers in Isfahan, Iran. Consistent with a study conducted in the U. S we found that motives for treatment-seeking varied across individuals [14]. The study uncovered three major themes including concerns with family, personal concerns with one’s quality of life mainly about one’s lifestyle and health, and economic concerns mainly about jobs and wealth. To our knowledge, this is the first study in Iran, and by extension, the Middle East that qualitatively analyzes core themes that motivated men with opioid dependence problems to seek treatment.

The participants in our sample were younger and older adult males with a mean age of more than 37 years and who reported that they entered themselves into OUD treatment services. In keeping with prior studies conducted in Canada [47], and the U. S [48, 49], the probability of seeking treatment for substance use disorders increase as people shift in adulthood. The reason is that adults are more concerned about the negative consequences of substance use disorders on their lives, and they obtain better benefits from treatment services compared to younger patients. Correspondingly, older patients are more likely to voluntarily seek treatment services and less likely to need social or family coercion for treatment-seeking.

Many participants in our study emphasized the importance of damage to one’s quality of life due to OUDs in the decision to seek treatment. Concerns over adverse effects of illicit opioid use on patients’ quality of life were repeated the most among all identified sub-themes. Significant damage to lifestyle was a serious fear of patients. This finding converges with prior studies from other countries like Turkey, India, the U. S, and Australia referring to “recognition of the negative effects of substance use on a patient’s own life” [30], “disgusted with oneself” [14], “having become a habitual user” [50], “lack of respect” [50], “realizing that misusing opioids was no longer an effective coping mechanism” [17], and housing problems [51]. Consistent with the literature [41, 51], patients in our sample reported the decision to seek treatment to recover from adverse physical and psychiatric consequences of opioid use. It is well-documented that opioid use is associated with physical and psychiatric diseases [16, 17, 19, 40]. As a notable example, a study which collected the required data from three residential facilities delivering integrated substance abuse and mental health services in Memphis, Tennessee, Malibu, California, and Palm Springs, reported that opioid users are at the highest risk of mortality among other illicit drug users [15]. On the other hand, withdrawal symptoms cause more physical and psychiatric disorders and exacerbate patients’ fears and concerns [8, 40].

It was a curious finding that only two participants referred to wishing for crime avoidance as a motivator for seeking treatment. There is a substantial social stigma associated with criminal activities in Iranian society. Also, crime is attached to drug use from an Iranian perspective. Keeping all of this in mind, it seems that participants might be concerned about profound impairments of their dignity due to being known as criminal drug users, and hence they hesitated to talk about their fears related to criminal activities. Only two participants said they sought treatment services because they were concerned about the opioid injection crisis. The opioid injection is not popular in this country, and that was consistent with our sample. Only those two participants who stressed their concerns about injection consequences had injected opioids before seeking treatment service.

The concern with family was the other significant motivation leading opioid patients to decide to seek treatment. Concern about upsetting family relationships was the most repeated sub-theme in this global theme. This finding is consistent with the literature [14, 16, 40, 52], which supports the importance of family-related motivators in seeking treatment. OUD usually destroys mutual patient and family trust. It is very important to help both patients and their families recover mutual trust.

As a notable finding, few participants reported family persuasion as a motive for seeking treatment services. It was shown in a prior study that verbal persuasion by a family member or a partner might not be an influential motivator for seeking treatment among people with substance use disorders [14]. Most participants in our study sample had experienced prior unsuccessful treatment therapies. Those unsuccessful experiences might cause hopelessness in patients’ families over substance use disorder treatment. It is possible that a patient’s family might consider treatment programs for substance use disorders ineffective. Other concerns with low citation included being “a negative role model for children.” Some studies in the U. S referred to fears of substance users (males or females) about the negative impacts of their behaviors on children [53–55]. Less than half of the participants in our study were fathers. Still, it seems that even most participants who were fathers were not worried about their paternal role modeling. One possible justification is that they might feet shame when talking about their damaged fathering. Further research is needed to investigate the association between paternity and motivation for seeking opioid use treatment.

Our findings also identified that concerns about financial difficulties, losing a job, and job reputation emerged as effective forms of motives for patients to seeking opioid treatment services. As previously mentioned the participants in our study were adult men and hence were of working age. Predictably, they might be worried about vocational difficulties, job loss, unemployment, and financial difficulties related to their substance use disorders. Therefore, economic concerns could persuade patients to seek treatment services. This is consistent with existing research. This finding supports other studies citing the effect of the economic consequences of OUDs on treatment-seeking. In a sample of 100 Indian male opiate users participating in a treatment program at a tertiary care hospital, it was found that 72 participants (72%) reported an inability to afford opiates as an important motivation for engagement in treatment [5]. In another study in India, a sample of patients (mostly opioid users) reported that they were “almost bankrupt,” it was “difficult to manage finances at home,” and they were “dismissed from a job due to substance use” as reasons for encampment in substance use treatment services [50].

Participants in this study reported their desire to resolve occupational failures by withdrawing from substance use disorders. Depriving recovered individuals of job opportunities in the labor market due to their past OUDs could exacerbate patients’ fears and deter them from seeking help for treatment-seeking. It seems that government and social outreach efforts are needed to promote incentives of potential treatment seekers for joining treatment services.

To sum up, the majority of interviewees thought that personal impact was more salient in their attempts for treatment. A minority of participants, however, stated that family-induced motivations were the main reasons behind their treatment decision. The results of the current study could offer policymakers and clinical therapists potential suggestions throughout the treatment process. Participants reported different motives for seeking treatment. A better understanding of perceived fears and concerns that acted as motives for seeking treatment will be critical for providing appropriate treatment. Therefore, innovative treatment procedures could and should be implemented concerning each patient’s motives for seeking treatment. Treatment methods need to target patients’ concerns and fears to achieve higher levels of treatment adherence and better treatment outcomes.

### Study limitations

First, a range of eight treatment centers providing various treatment services was selected as sampling strata. They included residential, in-patient, and out-patient centers that provided maintenance or sustenance treatments. This method of sampling helped us reach data saturation. However, as our sampling strategy in the selection of study centers was a convenience sampling method, there is a possibility that, despite a relatively large sample size of participants, selected centers and participants might not reflect the scope of the treatment seeking motives in its true richness and depth across the society. Accordingly, one possibility in future studies might be to select participants according to pre-defined and specific motives (e.g. motives revealed in the current study) in order to investigate them in a more detailed and rigorous fashion.

Second, we excluded female patients because of difficulties in accessing and persuading them to participate in a face-to-face interview. In fact, due to social stigma and personal barriers, female patients are more hesitant to participate in substance abuse treatment centers and are less willing to speak about their experiences, fears, and concerns [25, 56]. Future research can contribute to the findings of this study by exploring motives for treatment-seeking female patients with OUDs. This can help clinicians and policymakers adapt treatment strategies based on gender differences in motives.

The third limitation was related to response bias due to social desirability. This bias refers to the participants’ tendency to admit to socially desirable or acceptable thoughts and to deny socially undesirable ones rather than truthfully reporting their true thoughts [57]. To minimize this bias we collected data through a mechanism that secured participants’ privacy by conducting interviews in private rooms while ensuring them that their information will be kept confidential and anonymous [58]. Finally, due to the qualitative thematic method of the study, motives for seeking treatment were extracted through interviewing patients and analyzing their quotations. However, as cited in a study [26], a thorough understanding of motives leading patients to seek treatment needs further research based on observational studies of patients’ behaviors.

### Implications for policy and treatment

1. To develop the best treatment strategy, therapists need to consider patients’ motivations to refine their lifestyle. Patients also need professional advice about how to change bad their habits and harmful behaviors. The inclusion of behavioral therapy in treatment programs would persuade patients to enter and remain in the program.
2. Therapists need to add health care services to the treatment programs to alleviate withdrawal symptoms and persuade patients to remain in the treatment program.
3. Considering patients’ wishes to rebuild family relationships, therapists could integrate family support into the treatment procedure. In fact, family consultation should be a vital part of any treatment program.
4. The treatment strategies could target to raise family awareness about the long-lasting process of substance use therapy. The treatment strategies also need to target the family’s active engagement in promoting patients into treatment services.
5. Occupational benefit policies targeting patients in treatment programs can improve patients’ attitudes toward treatment-seeking.
6. Adapting labor laws and raising public awareness about the substance recovery process can show potential treatment seekers positive signs of a more equal work environment in response to recovery. This matter can also give them a positive attitude toward resolving economic difficulties through recovery and hence raise their possibility of joining treatment services.

## Conclusions

Our study highlights that men with OUDs have different motivations for seeking treatment. Using a qualitative method, we uncovered three global motivational themes from the perspective of patients who entered themselves into opioid use treatment. These included motives related to one’s quality of life, followed by motives related to family and economic motives. These findings could help policymakers and treatment providers to better understand opioid-use patient’s perceived concerns and fears as motives for treatment-seeking.

## Data Availability

The datasets used and/or analyzed during the current study are available from the corresponding author on reasonable request.

## Abbreviations

DALYs: Disabilty Adjusted Life Years
DIC: Drop-In Center
IDUs: Injection Drug Users
ICD: International Classification of Disease
MMT: Methadone Maintenance Treatment
OUD: Opioid Use Disorder
